# Case report: Seropositive myasthenia gravis complicated by limbic encephalitis positive for antibodies to AMPAR and Lgi1

**DOI:** 10.3389/fneur.2023.1237140

**Published:** 2023-10-12

**Authors:** Magne Solberg Nes, Mette Haugen, Hans Kristian Haugland, Nils Erik Gilhus, Christian Alexander Vedeler

**Affiliations:** ^1^Department of Neurology, Haukeland University Hospital, Bergen, Norway; ^2^Department of Pathology, Haukeland University Hospital, Bergen, Norway; ^3^Department of Clinical Medicine, University of Bergen, Bergen, Norway

**Keywords:** myasthenia gravis, autoimmune encephalitis, neuroimmunology, immunology, thymoma

## Abstract

**Objectives:**

Autoantibodies to the α-amino-3-hydroxy-5-methyl-4-isoxazolepropionic acid receptor (AMPAR) and leucine-rich glioma-inactivated 1 (Lgi1) are associated with autoimmune encephalitis. We described an acetylcholine receptor (AChR)-positive patient with myasthenia gravis who developed limbic encephalitis with antibodies to AMPAR and Lgi1.

**Methods:**

A single-case report with detailed, prospective clinical and biomarker data including serial laboratory testing and histopathology.

**Results:**

A 49-year-old woman was diagnosed with anti-AChR antibody-positive generalized myasthenia gravis in 1983. After 9 months of the removal of thymoma in 1984, she developed influenza-like symptoms and then symptoms of limbic encephalitis. Retrospective analysis of serum showed high concentrations of anti-AMPAR and lower concentrations of anti-Lgi1 antibodies. Cerebral CT was normal, EEG showed bifrontal dysrhythmia, and CSF showed mild pleocytosis. Immuno-histochemical examination of the thymoma confirmed staining for Glur2, a subunit of AMPAR. The patient recovered with mild sequelae, but low levels of anti-AMPAR and anti-Lgi1 antibodies were detectable for over 25 years subsequently.

**Discussion:**

This case confirms earlier reports of AMPAR-associated autoimmune encephalitis co-occurring with thymoma and myasthenia gravis and is unique in its observational length. It shows, moreover, that antibodies to AMPAR and Lgi1 can persist despite clinical recovery.

## 1. Introduction

Generalized myasthenia gravis is commonly associated with autoantibodies targeting the acetylcholine receptor (AChR) and, in some patients, it is associated with either thymus hyperplasia or the presence of a thymoma. The latter can also be associated with a broad array of neuroglial antibodies linked to autoimmune encephalitis (1).

We present a retrospective, single-case report of generalized myasthenia gravis with thymoma complicated by limbic encephalitis several months following the initial treatment of myasthenia. Retrospective serum testing showed high levels of autoantibodies targeting the α-amino-3-hydroxy-5-methyl-4-isoxazolepropionic acid receptor (AMPAR) and lower levels of antibodies to the leucine-rich glioma-inactivated 1 (Lgi1). Despite recovery, this patient continued to have low levels of neuroglial autoantibodies that did not appear to be associated with a higher risk of relapse.

## 2. Case report

A previously healthy 49-year-old woman was diagnosed with myasthenia gravis with elevated levels of antibodies to the AChR in 1983. Investigation revealed a thymoma, and she underwent a thymectomy in 1984 with a complete macroscopic removal of the thymus and thymoma. Histological examination showed a focal lymphoepithelioma in an otherwise normal thymic gland. She was treated with a combination of oral prednisolone and azathioprine as well as pyridostigmine. She also received a single series of plasma exchanges following the thymectomy to prevent disease relapse.

After 9 months of thymectomy, she experienced influenza-like symptoms and within days, she developed cognitive decline with amnesia that progressed over the course of a few days. On hospital admission, she was fully alert and cooperative but had both anterograde and retrograde amnesia. Neurological examination was otherwise normal. Neuropsychological assessment demonstrated marked defects in information retention, learning, and spatial orientation. Cerebral CT was normal, but EEG showed episodes of bilateral rhythmic frontal and temporal 2–5 Hz activity. The cerebrospinal fluid showed slight pleocytosis (11 mononuclear cells/mm^3^, normal <5/mm^3^), normal IgG concentration, and no oligoclonal IgG bands. On the presumptive diagnosis of autoimmune encephalitis, she was treated with plasma exchange. Her cognitive symptoms improved dramatically although her antegrade affection persisted for approximately 6 months. The following year she had a recurrence of amnesia affecting short-time memory that improved markedly with plasma exchange. Subsequently, she remained well and was able to live independently despite mild memory difficulties.

**Figure 1:**
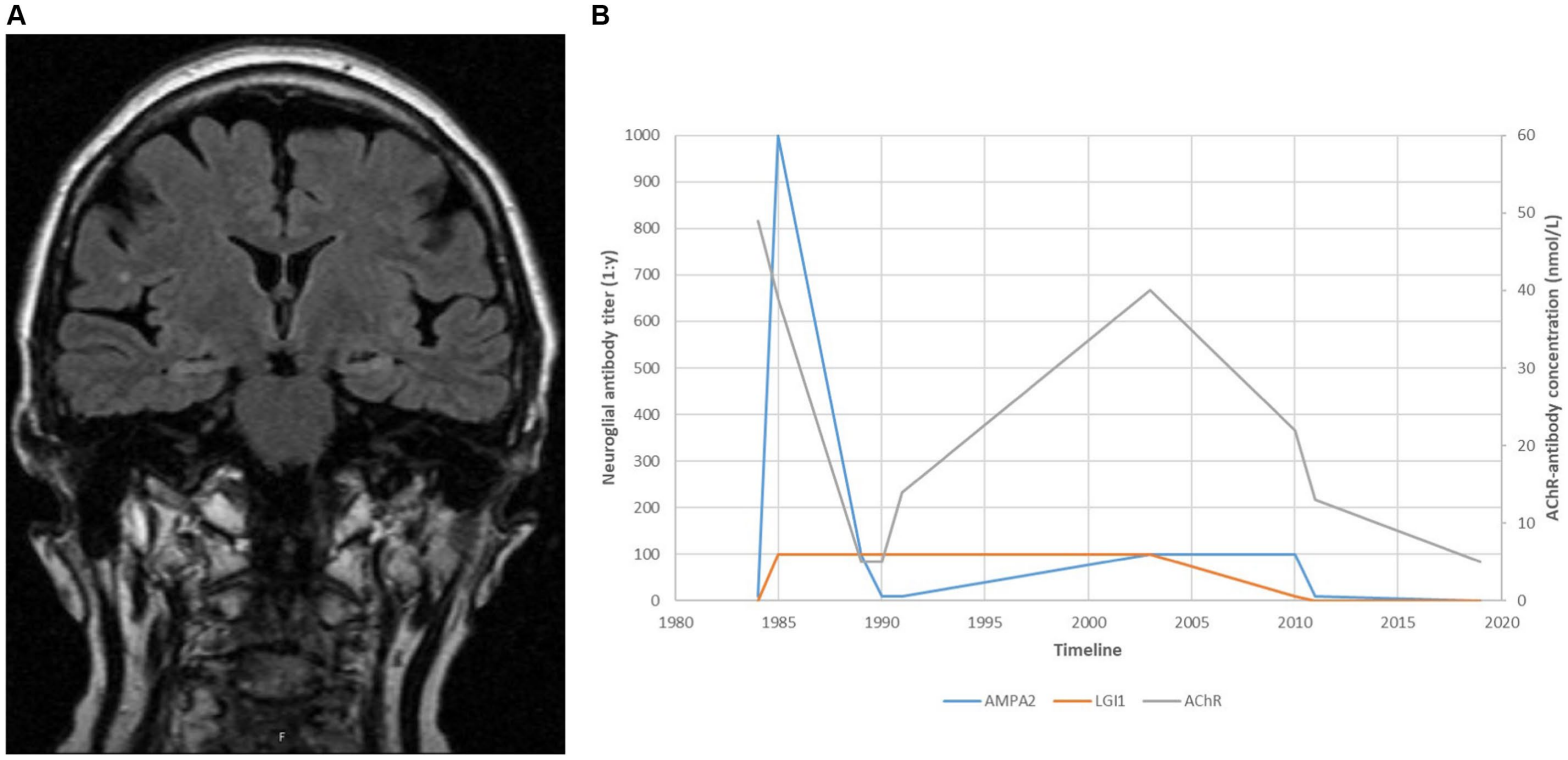
**(A)** A cerebral MRI taken in 2005, suggestive of medial temporal sclerosis approximately 20 years after recovery from distinct amnesia and both ante- and retrograde affection. **(B)** Antibody titres over time.

Her myasthenic symptoms remained well controlled apart from minor fluctuations. In 2002, her weakness worsened, and the serum levels of AChR antibodies increased following a period of poor medical compliance. Her symptoms remained stable following the reintroduction of azathioprine. Cerebral MRI in 2005 was suggestive of bilateral hippocampal sclerosis (Figure 1A).

We retrospectively tested the patient’s serum samples using immunoblots from Ravo Diagnostica GmbH^1^ and cell-based assays from Euroimmune AG^2^ for comparison. Samples collected during the first encephalitis episode in 1984 showed high levels of AMPAR antibodies and lower concentrations of antibodies to Lgi1. The AMPAR antibody level increased sharply during the recurrence of limbic encephalitis in 1985 and declined following plasma exchange. Both anti-AMPAR and anti-Lgi1 antibodies persisted in low concentrations for the next 25 years (Figure 1B).

**Figure 2:**
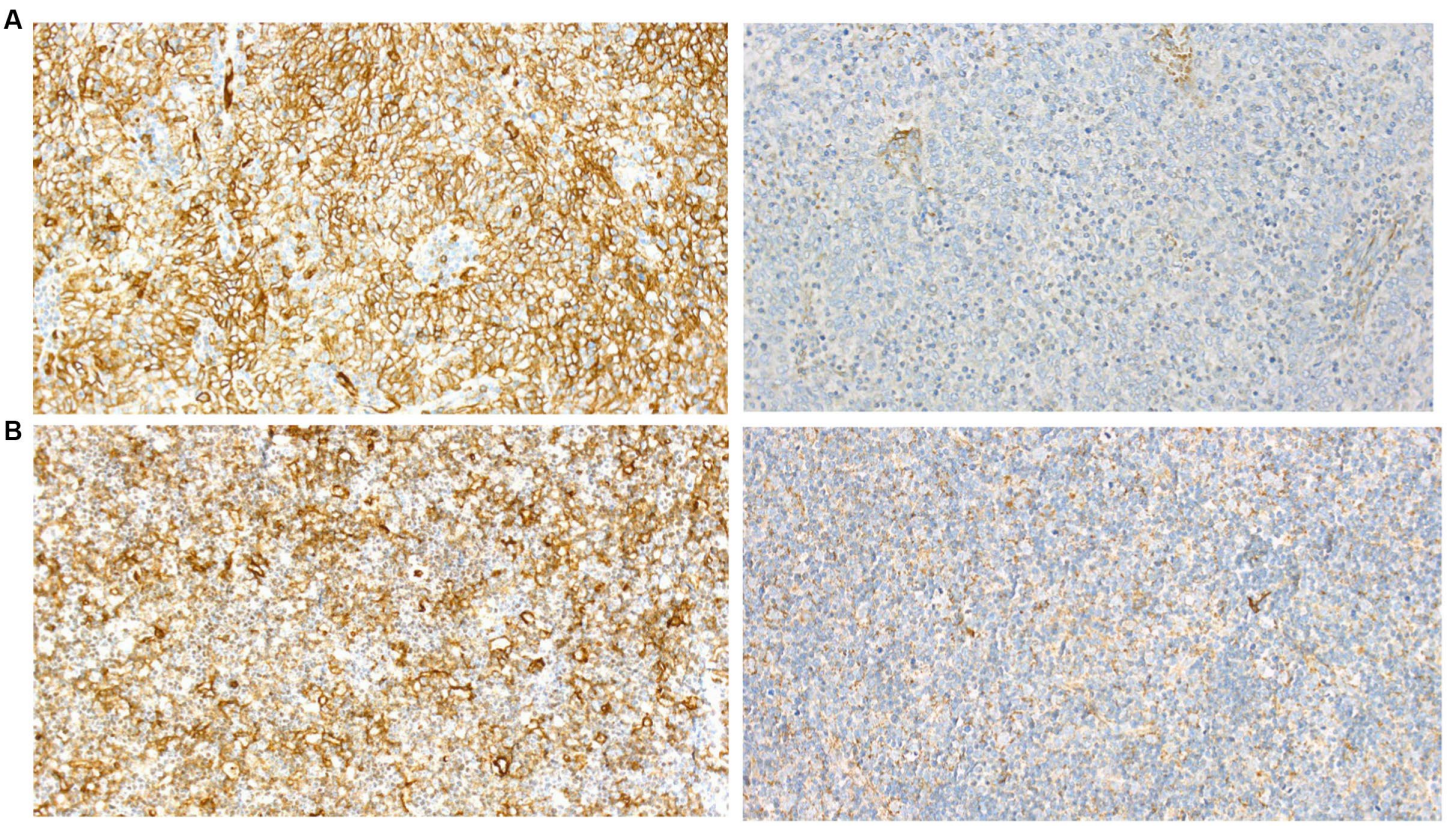
Immunohistochemical examination of thymoma from **(A)**, our patient and **(B)**, a control for GluR2 (left) and Lgi1 (right) showing similar staining pattern.

Immunohistochemical analysis of the thymoma showed strong positive staining for the GluR2-subunit of the AMPAR (Anti-GluR2 Mouse Monoclonal Antibody, Invitrogen, #32–0300) and moderate staining for Lgi1 (Anti-LGI1 Rabbit Antibody, Sigma Aldrich, #PRS4489) (Figure 2). Lymphoepitheliomas from three patients with myasthenia gravis with no encephalitic serum antibodies and not suffering from encephalitis were tested with the same antibodies, and all showed similar staining.

## 3. Discussion

This case was first reported in 1989 (2) and re-studied recently with testing of neuroglial antibodies targeting an AMPAR subunit and Lgi1. The clinical onset of limbic encephalitis occurred 9 months after the removal of the thymoma and was directly preceded by symptoms of a viral infection. Based on the serological levels of the two antibodies and a clinical picture dominated by symptoms of limbic encephalitis without faciobrachial dystonic seizures, we suspect the probable causative antibody to be against AMPAR.

While earlier case reports have described the co-occurrence of thymomas, myasthenia gravis with AChR-antibodies, and autoimmune encephalitis associated with both AMPAR and Lgi1 antibodies (3–5), the pathological mechanisms underlying this association remain unknown. A recent study found an increased risk of autoimmune disease following thymectomy (6), and some patients with thymoma-related myasthenia gravis can experience symptom worsening and an increase in anti-AChR antibody titers following thymectomy (7, 8). This was not, however, found in our case.

Retrospective analysis showed that our patient had high titers of anti-AMPAR and anti-Lgi1 antibodies when she first presented with limbic encephalitis in 1985, but low concentrations (1:10) of the AMPAR antibody were also present the preceding year when she was hospitalized for myasthenia gravis. Low concentrations of such antibodies do not occur frequently in the normal population. This suggests that the increased levels of these antibodies were not a result of the thymectomy but rather that the removal of the thymus and thymoma together with an infection activated an already established pathological immune response.

The patient’s thymoma stained strongly for the AMPAR-subunit GluR2 and to a lesser degree for Lgi1. We found a similar staining pattern in three thymomas from patients with no encephalitic serum antibodies suggesting that AMPAR and GluR2 represent self-antigens in the thymoma as part of negative T cell selection. Whether the AMPAR and GluR2 antigens in thymomas associated with autoimmune encephalitis differ from those present in subjects without autoimmunity is unknown, we know that patients with paraneoplastic cerebellar degeneration have genetic alterations in Yo-antigens in their ovarian tumors (9). We know, moreover, that HLA predisposition for autoimmunity is important as this has been linked to abnormal T cell autoreactivity (10).

Our analysis of patient samples collected over a 25-year period represents the longest longitudinal observation of AMPAR and Lgi1 antibodies documented. Our findings highlight the potential risk of multiple autoantibody associations, including that between limbic encephalitis and thymoma. In our patient, anti-AMPAR antibody levels increased after thymectomy, and a viral infection could have been a precipitating factor for limbic encephalitis. Following recovery, low levels of autoantibodies could be detected for 25 years without any signs of clinical progression. This suggests that low levels of these antibodies are not an indication of ongoing autoimmune encephalitis, but rather a marker of a previous episode. This contrasts with recent findings where persisting autoantibodies to the N-methyl-D-aspartate receptor (NMDAR) in patients with NMDAR-encephalitis were associated with a higher risk of relapse (9).

## Data availability statement

The raw data supporting the conclusions of this article will be made available by the authors, without undue reservation.

## Ethics statement

Ethical review and approval was not required for the study on human participants in accordance with the local legislation and institutional requirements. Written informed consent from the patients/participants or patients/participants’ legal guardian/next of kin was not required to participate in this study in accordance with the national legislation and the institutional requirements. Written informed consent was obtained from the individual(s) for the publication of any potentially identifiable images or data included in this article.

## Author contributions

MN, CV, and NG contributed to the conception and design of the study. MH organized the database. HH contributed to histopathological data. All authors contributed to the manuscript revision, and read and approved the submitted version.

## Footnotes

^1^www.ravo.de

^2^www.euroimmun.com

## In memoriam

In memorial of Prof. Johan Arild Aarli (1936–2023) who initially took care of the patient. Prof. Aarli was a close friend, colleague, mentor, and president of the World Federation of Neurology 2005–2009.
